# Production of Outer Membrane Vesicles by the Plague Pathogen *Yersinia pestis*

**DOI:** 10.1371/journal.pone.0107002

**Published:** 2014-09-08

**Authors:** Justin L. Eddy, Lindsay M. Gielda, Adam J. Caulfield, Stephanie M. Rangel, Wyndham W. Lathem

**Affiliations:** Department of Microbiology-Immunology, Northwestern University Feinberg School of Medicine, Chicago, Illinois, United States of America; Tulane University School of Medicine, United States of America

## Abstract

Many Gram-negative bacteria produce outer membrane vesicles (OMVs) during cell growth and division, and some bacterial pathogens deliver virulence factors to the host via the release of OMVs during infection. Here we show that *Yersinia pestis*, the causative agent of the disease plague, produces and releases native OMVs under physiological conditions. These OMVs, approximately 100 nm in diameter, contain multiple virulence-associated outer membrane proteins including the adhesin Ail, the F1 outer fimbrial antigen, and the protease Pla. We found that OMVs released by *Y. pestis* contain catalytically active Pla that is competent for plasminogen activation and α2-antiplasmin degradation. The abundance of OMV-associated proteins released by *Y. pestis* is significantly elevated at 37°C compared to 26°C and is increased in response to membrane stress and mutations in RseA, Hfq, and the major Braun lipoprotein (Lpp). In addition, we show that *Y. pestis* OMVs are able to bind to components of the extracellular matrix such as fibronectin and laminin. These data suggest that *Y. pestis* may produce OMVs during mammalian infection and we propose that dispersal of Pla via OMV release may influence the outcome of infection through interactions with Pla substrates such as plasminogen and Fas ligand.

## Introduction

Outer membrane vesicles (OMVs) are closed spherical portions of the bacterial outer membrane that contain phospholipids, outer membrane proteins, lipopolysacharide (LPS), and periplasmic contents [1]. Produced by many Gram-negative bacteria such as *Escherichia coli*, *Pseudomonas aeruginosa*, and *Helicobacter pylori* [2]–[4], OMVs are formed when small portions of the outer membrane pinch off from the cell and are released as self-contained spherical structures that range from 20–250 nm in size [5]. While the biogenesis of OMVs is poorly understood, it is thought that expansion of the outer leaflet of the membrane relative to the inner leaflet induces membrane curvature that forces the outer membrane to bud away from the cell [5], [6]. OMV production can be detected in bacterial communities growing under a variety of conditions, including planktonic cultures as well as in surface-attached biofilm communities [7], [8].

OMVs are produced by both pathogenic and non-pathogenic bacteria [9]–[11]. OMVs released by pathogens can contain multiple components that interact with the host, including LPS, virulence factors, and other antigens. Pathogen-derived OMVs may contribute to virulence by modulating the innate immune response, delivering toxins to cells, dispersing antigens and virulence factors away from the bacterium, trafficking signaling molecules between bacteria, and more. Microscopic examination of tissues has detected the presence of OMVs near host cells or within host tissues, suggesting an interaction between OMVs and the host during infection [12]–[14]. Further, OMVs have been found to deliver active toxins to host cells, including the enterotoxigenic *E. coli* heat-labile enterotoxin (LT), the enterohemorrhagic *E.coli* pore-forming cytotoxin ClyA, and the *H. pylori* VacA protein [3], [11], [15]. Environmental stresses contribute to the production of OMVs [16], suggesting that, as bacteria encounter stressors such as those found within the infected host, the production of OMVs may not only manipulate interactions with the host but also aid in the survival of the bacterium.

The Gram-negative bacterium *Yersinia pestis*, a pathogen of both insects and mammals, can be transmitted to humans via the bite of hematophagous insects (typically fleas) or through the inhalation of respiratory droplets or aerosols containing the bacteria, and can cause bubonic, pneumonic, or septicemic plague [17]. Temperature is a major regulator of gene expression in *Y. pestis*, controlling both transcriptional and post-transcriptional responses [18], [19]. At lower temperatures (<25°C), *Y. pestis* produces factors that maximize survival and colonization in the flea, such as biofilms [20], while at higher temperatures (>30°C), the bacterium expresses genes required for mammalian infection, including the adhesin Ail, the F1 fimbrial antigen (Caf1), the outer membrane protease Pla, and the Yop-Ysc type III secretion system (T3SS) [21]–[24]. Thus, *Y. pestis* possesses a variety of virulence factors, including a number of outer membrane-associated factors, which are necessary for interacting with its hosts to ultimately cause disease.

Among these, the Pla protease is necessary for the progression of both bubonic and pneumonic plague, but is dispensable during septicemic plague [21], [25], [26]. Pla is known to cleave a number of mammalian host proteins, including the zymogen plasminogen (plg), the plasmin inhibitor α2-antiplasmin, and the recently identified substrate Fas ligand (FasL), a major inducer of host cell death via apoptosis [27]–[31]. In addition, Pla has also been shown *in vitro* to act as an adhesin to extracellular matrices by binding laminin as well as promoting the bacterial invasion of HeLa cells [24], [32], [33].

As Pla is an insoluble outer membrane protein dependent on rough LPS for its protease activity, it is not thought to be secreted by *Y. pestis* [34]–[36]. However, we have detected active Pla in cell-free culture supernatants, suggesting that this cell-free form of Pla could be contained on OMVs. Here we investigate the ability of *Y. pestis* to produce native OMVs, characterize the presence and activities of various virulence factors carried on released OMVs, and propose a role for these OMVs during mammalian infection.

## Results

### Outer membrane protein activity in cell-free culture supernatants

**Figure 1:**
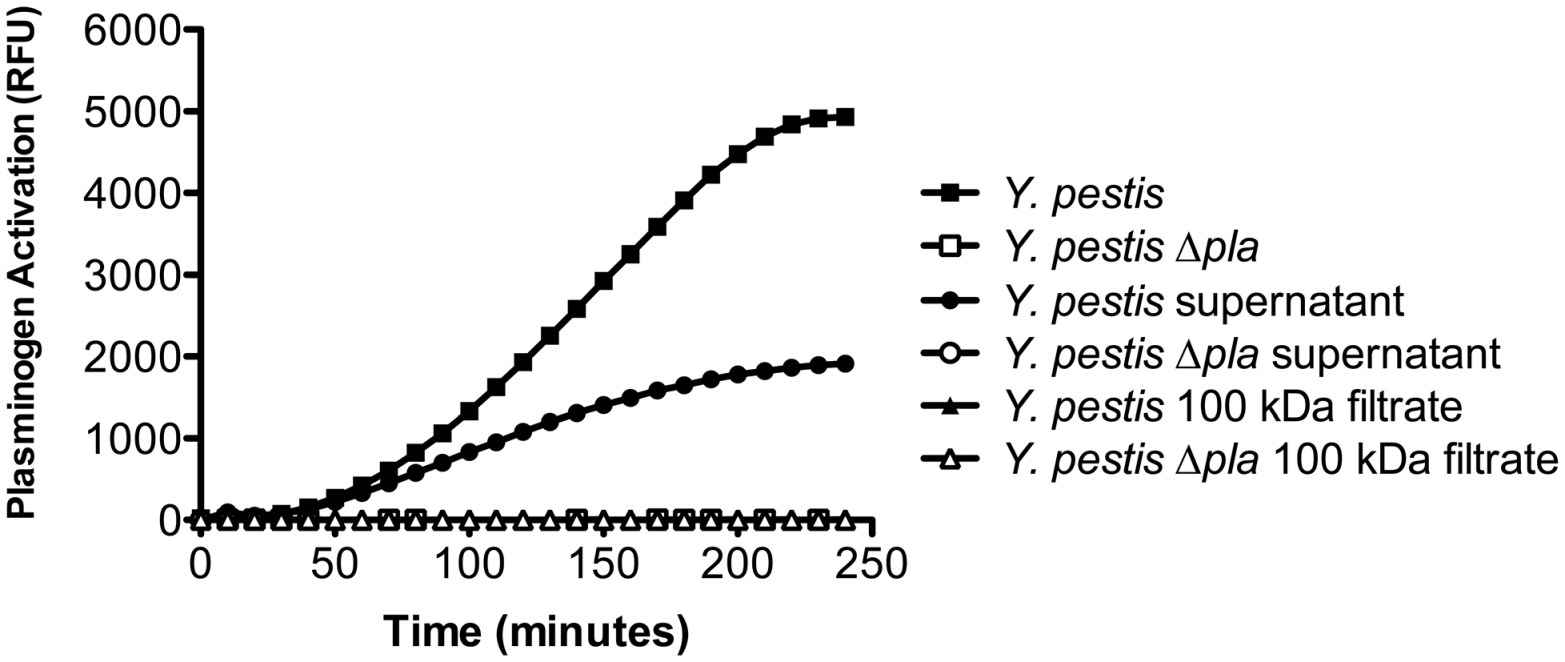
*Y. pestis* culture supernatants contain active Pla. (A) Plg-activating ability of whole bacteria, 0.2 µm-filtered culture supernatants, or the filtrate of 100 kDa-passed culture supernatants, from wild-type or Δ*pla Y. pestis*, respectively. Materials were incubated with human glu-plg and a fluorescent substrate of plasmin for 3 h at 37°C. One experiment representative of 3 independent biological replicates is shown.

Our laboratory has detected the activity of the outer membrane protein Pla in cell-free culture supernatants during the exponential growth phase of *Y. pestis.* To explore this further, 0.2 µm-filtered, cell-free culture supernatants from either wild-type *Y. pestis* or an isogenic mutant of *Y. pestis* lacking Pla (*Y. pestis* Δ*pla*) were grown in the rich media brain-heart infusion (BHI) at 37°C and tested for the ability to convert plg to the active plasmin form, an activity dependent on Pla. We found that filtered culture supernatants from wild-type but not *Y. pestis* Δ*pla* contained measurable levels of Pla activity (**Fig. 1**). This activity was lost when these 0.2 µm-filtered culture supernatants were further passed through a filter with a 100 kDa cutoff (**Fig. 1**). As the molecular weight of Pla is 37 kDa and Pla is not predicted to form multimers, these data suggest that the form of Pla found in cell-free culture supernatants may be contained on bacterial superstructures greater than 100 kDa [37]. While this could represent cellular lysis, the observation of OMV formation by other Gram-negative bacteria prompted the consideration of OMV production by *Y. pestis*.

### *Y. pestis* produces OMVs

**Figure 2:**
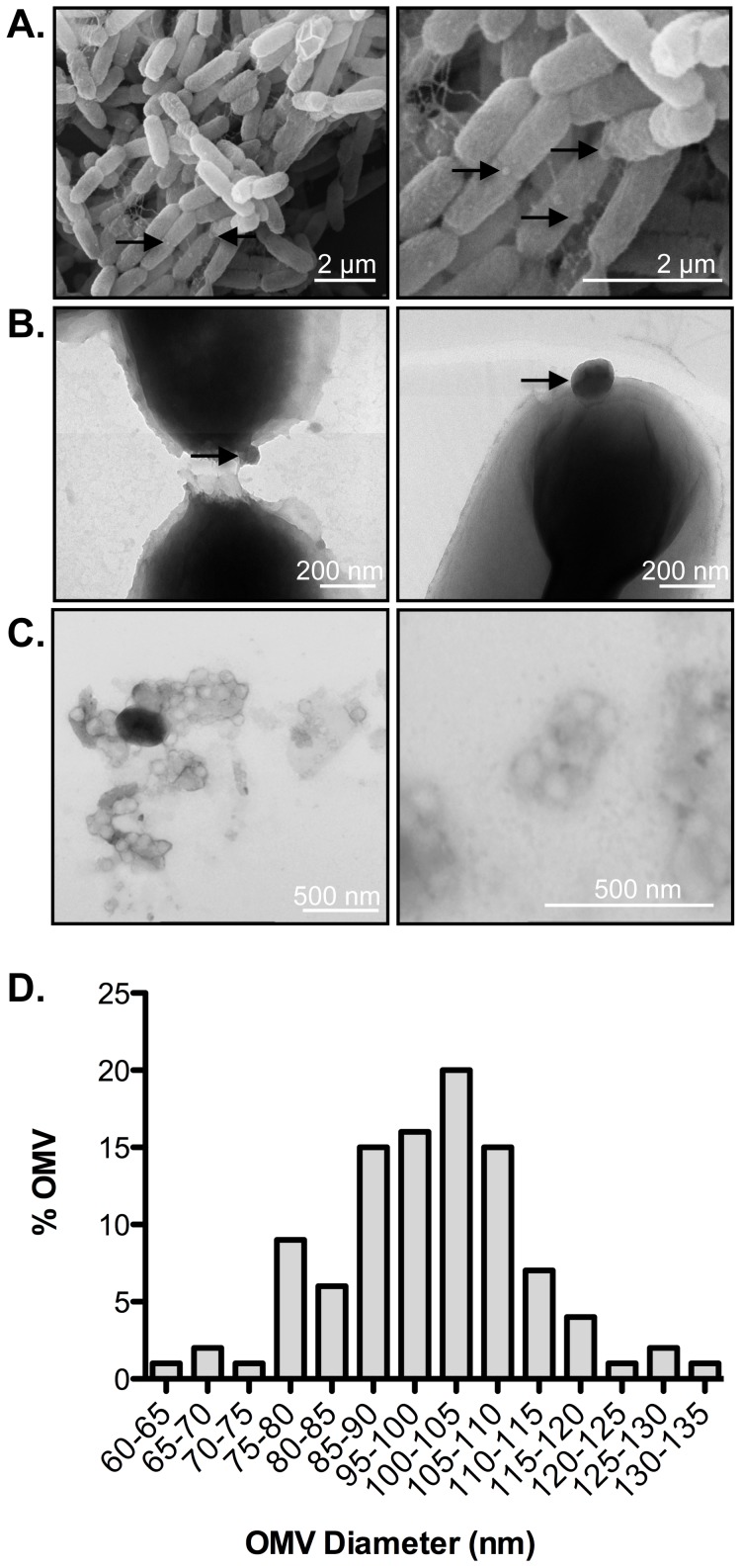
*Y. pestis* produces membrane blebs consistent with OMVs. *Y. pestis* bacteria cultured for 6 h at 37°C were fixed and imaged via SEM. (A and B) Images reveal round membrane protrusions on the bacterial surface (arrows) that are consistent with OMVs. (C) TEM of OMVs purified from *Y. pestis* supernatants. Bar represents size in nanometers. (D) Size distribution of OMVs purified from *Y. pestis*. One hundred OMVs were measured and diameters are shown as a percent of the total. The average OMV diameter is 93.07+/−11.75 nm. Bars represent size in nanometers as indicated.

OMV-like structures have been previously observed on the surface of *Y. pestis* bacteria [38]. To examine whether *Y. pestis* produces OMVs under laboratory conditions, bacteria were cultured in BHI at 37°C and at various times during growth, aliquots of bacteria were removed, fixed, and examined via both scanning and transmission electron microscopy. Micrographs revealed round, vesicle-like structures attached to or affiliated with the surface of *Y. pestis* bacilli (**Fig. 2A & 2B**). While these structures could be artifacts of the fixation procedure, they are similar to those observed on the surfaces of other bacterial species, suggesting the formation of OMVs [39].

To determine whether these structures are truly natural products of *Y. pestis* and share characteristics with OMVs produced by other bacteria, we purified potential native OMVs released by *Y. pestis* using standard, established vesicle isolation techniques that do not require sonication, shearing, or chemical treatments to induce vesicle production [5], [40]. Briefly, 0.2 µm-filtered, mid-log growth-phase culture supernatants were concentrated and ultracentrifuged to isolate outer membranes. To purify vesicles from cellular debris, the isolated material was subjected to Optiprep-based gradient ultracentrifugation, resulting in a separation of contaminating cellular proteins and the OMVs based on lipid content into multiple independent fractions [3]. We analyzed the fractions by transmission electron microscopy and found characteristic OMV-like spherical structures (**Fig. 2C**), similar to vesicles isolated from other Gram-negative bacteria [1], [8], [41]. Together, these SEM and TEM images indicate that *Y. pestis* releases material under standard laboratory conditions that is consistent with that of bacterial OMVs. We determined the average size of these isolated OMVs to be 93.07+/−11.75 nm in diameter (**Fig. 2D**).

### Characterization of proteins associated with *Y. pestis* OMVs

**Figure 3:**
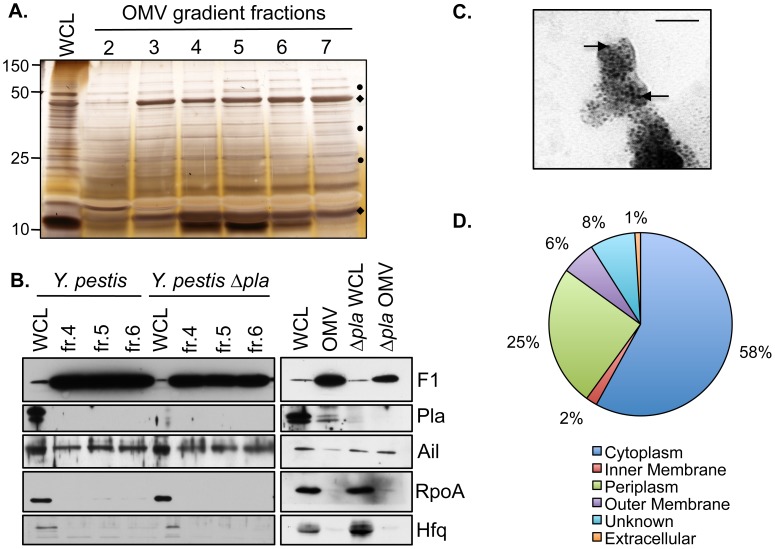
OMVs contain outer membrane-associated virulence factors. (A) Whole cell lysates (WCL) or density centrifugation gradient fractions from OMVs isolated from *Y. pestis* were separated by SDS-PAGE and gels were silver stained. (♦) denotes an enriched band and (•) denotes reduced bands. (B) WCL or gradient fractions (4–6) from OMVs isolated from *Y. pestis* and *Y. pestis* Δ*pla* were examined for the presence of the virulence factors Pla, Ail, and Caf1 by immunoblot. Immunoblots for RpoA and Hfq, two cytoplasmic proteins, are shown to demonstrate the absence of contaminating proteins from the OMV preparation. (C) OMVs were immuno-gold labeled with an anti-Caf1 antibody conjugated to gold beads and examined by TEM. Black arrows indicate representative gold particles. Bar represents 50 nm. (D) Subcellular distribution of proteins present in *Y. pestis* OMVs as a percentage of total proteins identified by mass spectrometry listed in (Table S3).

OMVs are known to carry a wide array of proteins associated with the outer membrane, periplasm, and cytoplasm of Gram-negative bacteria. Therefore, we examined if *Y. pestis* OMVs are enriched for protein subsets compared to whole bacteria and if specific outer membrane virulence factors are present on these OMVs. To minimize contamination from cellular lysis, OMVs were isolated from mid-log phase cultures without the use sonication or chemical treatment in order to purify naturally occurring OMVs. First, we analyzed by reducing SDS-PAGE fractions 2–7 of the Optiprep gradient used to purify OMVs. We found that fractions from the Optiprep gradient contained proteins that were either enriched or reduced in abundance compared to *Y. pestis* whole cell lysates (WCL) (**Fig. 3A**). In *Y. pestis*, a number of proteins contained within or associated with the outer membrane are virulence determinants, and many of these are produced at 37°C, including Ail, Pla, and Caf1 [42]. Therefore, to determine if OMVs produced by *Y. pestis* at 37°C contain these specific outer membrane-associated proteins, immunoblot analyses were performed with antibodies to Ail, Caf1, and Pla. To determine the enrichment of these proteins compared to the cytoplasmic fraction, we also examined OMVs for the presence of Hfq, a cytoplasmic protein that serves as a chaperone for small RNAs, and RpoA, the alpha subunit of RNA polymerase that is also found in the cytoplasm. We consistently found that the outer membrane-associated proteins Ail and Caf1 were present in gradient fractions 4–6, and that these same fractions contained minimal RpoA and Hfq (**Fig. 3B**, left panels). On the other hand, we were unable to detect Pla in the individual pure OMV fractions; therefore, to increase protein abundance we combined and concentrated fractions 4–6 and repeated the same immunoblot analysis. Using this approach we could detect the presence of Pla on the OMVs (**Fig. 3B**, right panels). We also isolated OMVs from the Δ*pla* strain of *Y. pestis*, as Pla is known to cleave *Y. pestis* proteins and thus could alter the composition of the OMVs themselves [37], [43]. OMVs from *Y. pestis* Δ*pla* contain the outer membrane proteins Ail and Caf1 and lack Pla as well as the cytoplasmic proteins RpoA and Hfq (**Fig. 3B**, left panels). This indicates that the loss of Pla does not impact the presence or absence of these other *Y. pestis* proteins contained on OMVs. To demonstrate the presence of Caf1 on the surface of OMVs, OMVs were immuno-labeled with antibodies to Caf1 using 6 nm-sized gold beads. Transmission electron micrographs of OMVs labeled with anti-Caf1 antibody demonstrates that Caf1 protein is indeed present on the surface of isolated OMVs (**Fig. 3C**).

### Proteomic analysis of *Y. pestis* OMVs

In order to more thoroughly analyze the proteins associated with *Y. pestis* OMVs, we purified OMVs from bacteria cultured at 37°C in biological triplicate and analyzed the protein content by mass spectrometry. A total of 270 unique proteins present in at least 2 of the 3 replicates were identified and the subcellular localization of each protein was predicted using the PSORTb algorithm (**Table S1**). This analysis indicated that of the 270 proteins identified, 15 (6%) are derived from the outer membrane (including Ail and Caf1), 68 (25%) are found in the periplasm, 5 (2%) are from the inner membrane, and 160 (58%) are cytoplasmic (**Fig. 3D**). Of note, we failed to detect Pla peptides by mass spectrometry, even though we are able to observe the presence of Pla and its activity by immunoblot and other assays (see below). In total, these results confirm that native OMVs produced by *Y. pestis* contain and display a significant number of proteins, including multiple virulence factors.

### Increased production of OMVs in response to temperature and stress

**Figure 4:**
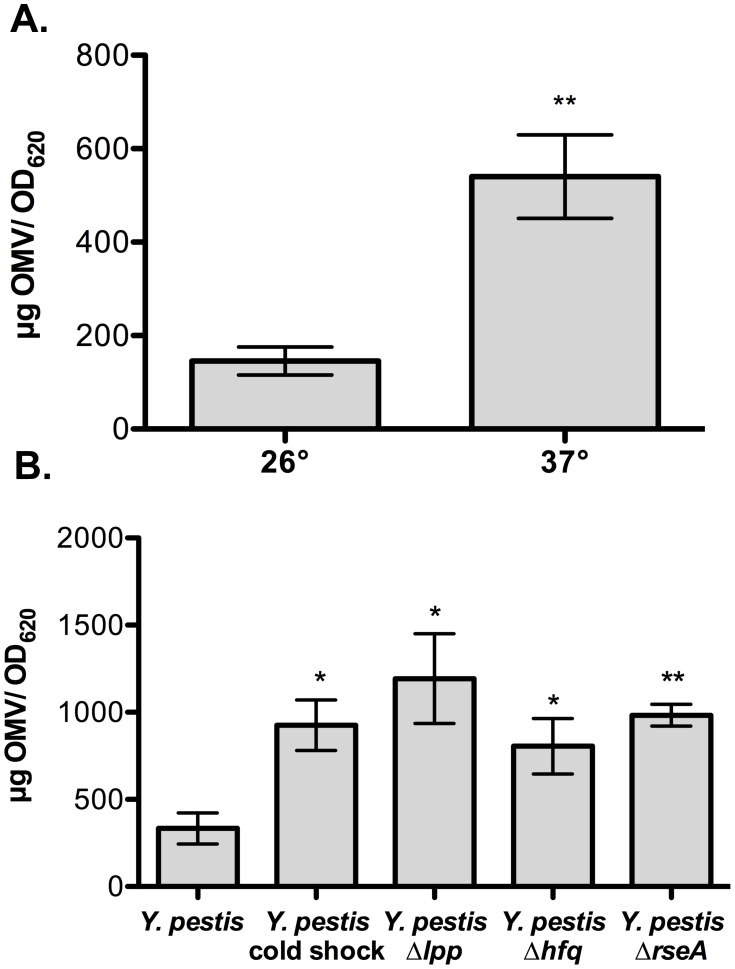
Effects of temperature and stress response factors on OMV production. (A) OMVs were isolated from *Y. pestis* cultured at either 26°C or 37°C, and total protein abundance associated with the OMVs was measured. Protein concentrations were normalized to the OD_620_ of the bacterial cultures. (B) Wild-type *Y. pestis* or strains lacking the genes for RseA, Hfq, or Lpp were cultured at 37°C as above and OMVs were isolated and total associated protein was measured and normalized to the OD_620_. For cold shock experiments, bacteria were placed in an ice water bath for one h before proceeding. One experiment representative of two biological replicates is shown. The mean and SE are shown. *p<0.05, **p<0.005 (student's t–test).

Temperature is a major regulator of gene expression in *Y. pestis*, and OMV production by other bacteria has been observed at both low and higher temperatures [7], [8], [44]. With this in mind, we examined whether changes in temperature affect OMV production by *Y. pestis*. OMVs were isolated from *Y. pestis* cultured at either 26°C or 37°C and total protein content associated with the purified OMV fractions was measured. We found a significantly greater quantity of OMV-associated protein released into the culture media at 37°C compared to 26°C, suggesting that OMV production is more abundant at elevated temperatures (**Fig. 4A**). In addition, activation of bacterial stress response pathways has been shown to increase the formation of OMVs [40], [45]–[48]. We first investigated the impact of cold shock, a well-established inducer of stress in Gram-negative bacteria [49], on OMV production by incubating cultures of *Y. pestis* grown at 37°C on ice for one hour. Quantification of OMV-associated proteins isolated from these cold-shocked bacteria demonstrated a significant increase in the release of OMVs compared to bacteria maintained at 37°C (**Fig. 4B**).

To test whether the loss of factors that respond to membrane stress contributes to or alters OMV production by *Y. pestis*, we employed deletions in the genes encoding RseA, Hfq, and the major Braun lipoprotein Lpp. The anti-sigma factor protein RseA is a negative regulator of SigmaE; deletion of *rseA* results in elevated activity of SigmaE, a regulator of the outer membrane stress response [50]–[52]. Hfq is a chaperone for small, non-coding regulatory RNAs (sRNAs), and recent studies have shown that Hfq is necessary for resistance to multiple stressors by *Yersinia* species [53]–[55]. Lpp links the outer membrane to peptidoglycan, and the deletion of *lpp* disrupts membrane stability, contributing to increases in OMV formation in several bacteria [5], [56], [57]. Isogenic deletions of *rseA*, *hfq*, and *lpp* in *Y. pestis* results in 3.0, 2.4, and 3.6-fold increases in OMV-associated proteins present in the culture media, respectively, when compared to OMV production by wild-type bacteria at 37°C (**Fig. 4B**). In total, these results provide evidence that the production and release of OMVs by *Y. pestis* likely increases when undergoing both temperature and cell envelope stress in a manner similar to other Gram-negative bacteria.

### *Y. pestis* OMVs contain active Pla

**Figure 5:**
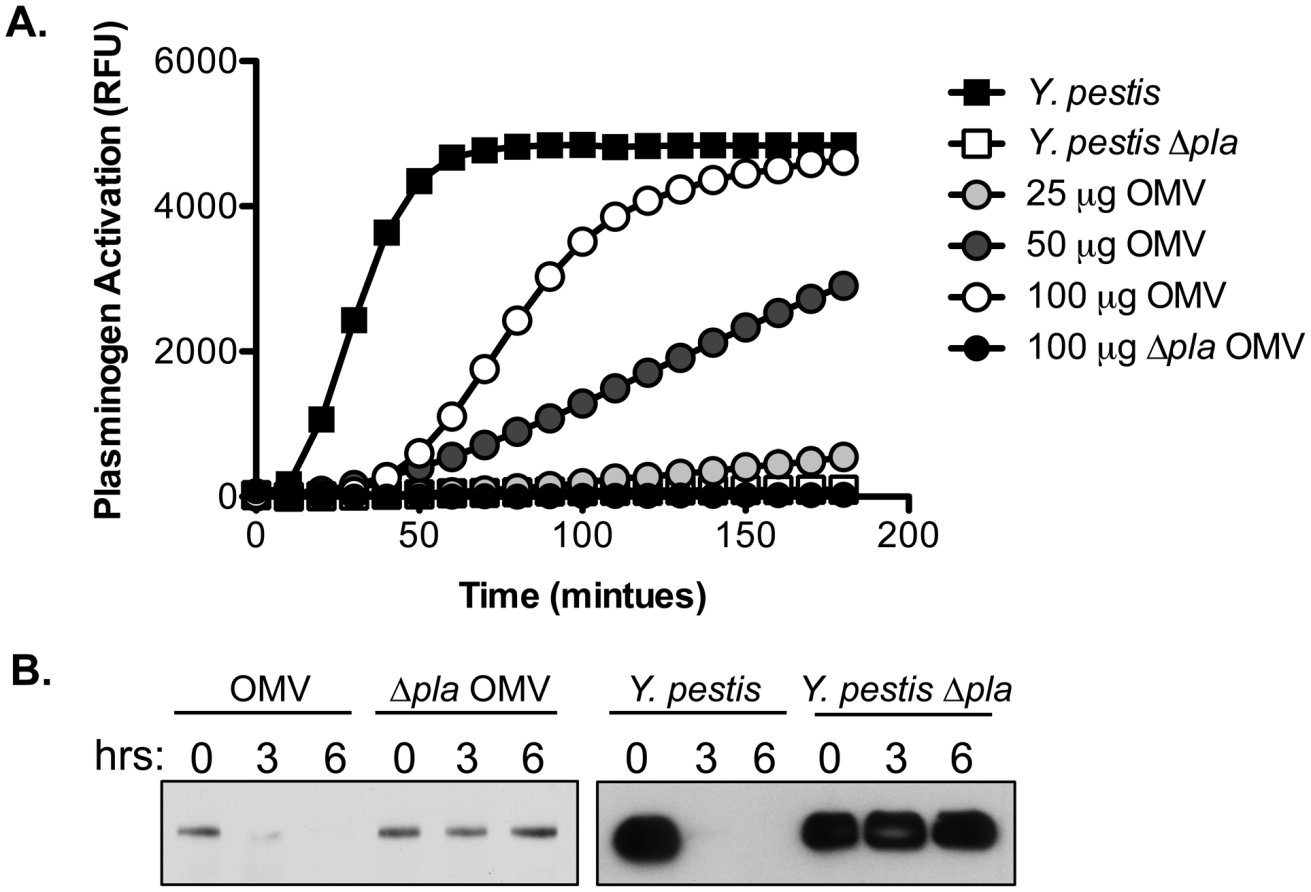
OMV-bound Pla is catalytically active and interacts with components of the ECM. (A) Plg-activating ability of wild-type or Δ*pla Y. pestis* bacteria or OMVs. Whole bacteria or purified OMVs were incubated with human glu-plg and a fluorescent substrate of plasmin for 3 hours at 37°C. (B) Degradation of α2-antiplasmin by wild-type or Δ*pla Y. pestis* bacteria or OMVs. Whole bacteria or purified OMVs were incubated with purified human α2-antiplasmin at 37°C and at the times indicated, the presence of uncleaved α2-antiplasmin was determined by immunoblot analysis.

Proteins contained within bacterial OMVs often retain biological activity [5], [58]–[60], therefore we hypothesized that OMV-bound Pla may remain catalytically active and able to cleave its substrates, such as plg. To test this, we isolated OMVs from wild-type and Δ*pla Y. pestis* and then performed a plg-activation assay with these vesicles. Wild-type OMVs containing Pla activated plg in a dose-dependent manner, while OMVs from *Y. pestis* Δ*pla* were unable to activate plg (**Fig. 5A**). We also examined the ability of purified OMVs to degrade α2-antiplasmin, another established substrate of Pla [28]. Incubation of OMVs with purified α2-antiplasmin resulted in a Pla-dependent loss of detectable α2-antiplasmin over time as determined by immunoblot analysis (**Fig. 5B**). Thus, these results demonstrate that OMV-bound Pla retains the ability to cleave known substrates of the protease.

### *Y. pestis* OMVs adhere to the extracellular matrix

**Figure 6:**
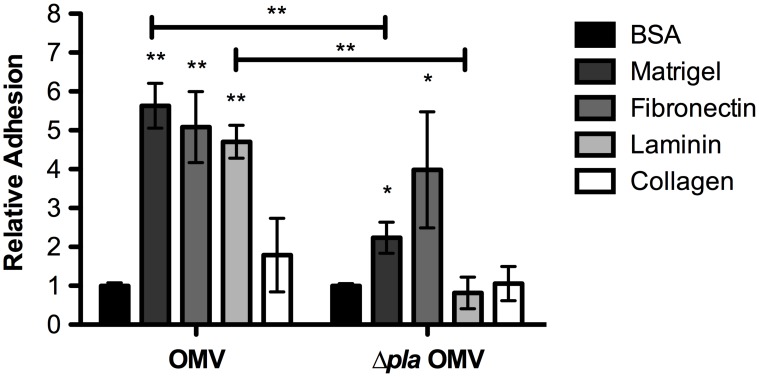
Binding of *Y. pestis* OMVs to components of the extracellular matrix. Wild-type or *Δpla* OMVs were examined by ELISA for the ability to bind the ECM components Matrigel, fibronectin, laminin, and collagen. BSA was used as a negative control for binding and differences in fluorescence are presented as fold change compared to BSA (set at 1). The combined mean and SE of 3 independent experiments are shown. *p<0.05, **p<0.005 (two-way ANOVA).

Both Ail and Pla facilitate binding of *Y. pestis* to components of the extracellular matrix (ECM) [61]. Since we have shown that *Y. pestis* OMVs contain these adhesins, we tested whether wild-type or Δ*pla* OMVs are also able to bind to ECM components. We incubated *Y. pestis*-derived OMVs in 96-well plates coated with Matrigel (a 3-dimensional ECM composed of laminin, collagen type IV, heparan sulfate proteoglycan, and entactin) or individual components of the ECM such as fibronectin, laminin, and collagen, and assessed binding by ELISA using fluorescently labeled anti-Caf1 antibodies. We found that OMVs derived from wild-type *Y. pestis* were able to interact with Matrigel, fibronectin, and laminin to a significantly greater degree than to bovine serum albumin (BSA)-coated wells, demonstrating that *Y. pestis* OMVs retain the ability to bind to ECM components (**Fig. 6**). Furthermore, the presence of Pla on these OMVs significantly contributes to the binding of OMVs to Matrigel and laminin, suggesting that in this context, Pla may also serve as an adhesin for OMVs to these components of the ECM (**Fig. 6**). Taken together, our data demonstrate that Pla retains both attributed biological functions (i.e adhesive and protease activities) when contained on OMVs.

## Discussion

A growing body of evidence suggests that OMVs play critical roles in the physiology and life cycle of many bacteria, including the killing of competing species, transferring genetic material to other bacteria, delivering toxins and virulence factors to host cells, and modulating the immune response of the host [3], [58]–[60]. While a large aspect of OMV research is aimed at understanding the host recognition of antigenic OMV-bound factors, particularly for vaccine development, the native activity of proteins on OMVs may also play distinct roles in pathogenesis and the modulation of host defense during bacterial infection. Indeed, this has been observed for enterotoxigenic *E. coli* via the OMV-mediated delivery of LT to host cells and for the OMV-mediated induction of IL-8 by *H. pylori* and *P. aeruginosa* [3], [4], [62].

Here we show that *Y. pestis*, the causative agent of plague, releases OMVs under physiological conditions. As expected based on their derivation, these OMVs carry multiple constituents of the outer membrane, although it is not yet known whether *Y. pestis* actively sorts specific proteins and/or modulates their abundance into OMVs during biogenesis. Our data suggest that a limited number of outer membrane proteins are associated with native OMVs produced by *Y. pestis in vitro* and that the F1 capsular antigen Caf1 represents the major constituent of the OMVs, as illustrated by immunoblot and a high MASCOT score determined by mass spectrometry. We hypothesize that the abundance of Caf1 might exclude other outer membrane proteins from associating with native OMVs. This is supported by our data indicating that there is less Ail per µg of protein in OMVs compared to the equivalent amount of protein derived from a *Y. pestis* whole cell lysate. This observation, coupled with the fact that Pla autoprocesses itself (potentially limiting the number of peptide fragments for detection), could explain why Pla protein levels were below the detectable limit in our mass spectrometry analysis, even though OMV-associated Pla activity can be measured in a variety of assays. Thus we cannot rule out that there may be additional proteins associated with OMVs that were not detected by mass spectrometry but could be identified through alternative protein isolation techniques.

Our analysis of the protein profile associated with native OMVs is consistent with a number of studies that find an abundance of both outer membrane and periplasmic proteins and an exclusion of inner membrane proteins [58], [60], [63]–[65]. Studies characterizing the outer membrane proteome of *Y. pestis* grown *in vitro* have shown that between 50 and 70 proteins are associated with the outer membrane and these are altered in a temperature shift between 26°C and 37°C [42], [66]. Additionally these studies identified 31 outer membrane proteins associated with the outer membrane fraction isolated from *Y. pestis* and we identified 15 outer membrane proteins by mass spectrometry that are associated with OMVs when bacteria are grown at 37°C, representing approximately 20% of the total outer membrane proteome and 33% of the OM proteins previously identified associated with whole bacterial membranes. We found a number of cytoplasmic proteins associated with *Y. pestis* OMVs, including Elongation factor Tu, GroEL, RpsA, RplL, and DnaK, which is consistent with findings from the studies of multiple Gram-negative OMVs, including *Neisseria meningitidis* [67], *E. coli* [65], *Brucella melitensis* [60], and *Edwardsiella tarda* [63]. Some of the cytoplasmic contaminants we observed are also known virulence determinants or immune stimulants such as GroEL, Ymt, and a tellurium resistance protein. These proteins were also identified as associated with *Y. pestis* OMV-like outer membrane blebs [38]. It is unknown whether these common cytoplasmic proteins associated with Gram-negative OMVs are contaminants or represent cytoplasmic proteins either non-specifically or specifically targeted into OMVs during their biogenesis.

Based on studies of OMV production by other bacterial pathogens during infection, we hypothesize that OMV release by *Y. pestis* in the mammal could have multiple physiological consequences, such as influencing the immune response to the infection, altering host cell function, and aiding bacterial spread through the dysregulation of the host hemostatic and innate immune systems. For instance, OMV interaction with the ECM could facilitate disruption of the epithelial layer via Pla or other factors, thereby permitting development of the characteristic edema and fluid accumulation observed during pneumonic plague. Indeed, OMVs from a variety of pathogens have been detected in the fluids of infected hosts, demonstrating their ability to spread from the site(s) of infection [68]–[70]. While it is not yet known if *Y. pestis* produces OMVs during infection, dispersal of OMVs may prove beneficial for the plague bacillus by delivering antigens and virulence factors, such as Pla or Ail, to sites distal to the bacterium. For instance, OMV-mediated dispersal of active Pla could expand the range of fibrin degradation near the bacteria, allowing for further bacterial spread in the tissue.

In addition, while the T3SS dampens immune cell activation around the bacteria themselves through the direct injection of T3SS Yop effectors into recruited hematopoietic cells, dispersal of OMVs could redirect the focus of polymorphonuclear cells away from the bacteria, prolonging bacterial survival. Furthermore, if OMVs interact with host cells that are not otherwise targeted by the T3SS, those host cells could themselves become activated in a manner that results in inflammation and further immune cell recruitment. We speculate that OMVs may allow for catalytically active Pla to act upon targets in the lungs during pneumonic plague, such as the newly discovered target Fas ligand, resulting in altered host cell apoptosis and innate immunity [29]. Thus, the production of OMVs by *Y. pestis* may provide an explanation for how a pathogen with a significant array of anti-inflammatory virulence factors is able to induce a highly pro-inflammatory state during disease.

*Y. pestis* infection of mammals is generally extracellular in nature, and the only bacterial products thought to be delivered to the host cell cytoplasm are those injected by the T3SS. It has been repeatedly demonstrated, however, that OMVs released by other bacterial pathogens are capable of fusing with or are internalized by host cells [4], [14]. Thus, OMV production by *Y. pestis* could potentially result in the delivery of otherwise extracellular or cell surface-associated bacterial factors directly to the eukaryotic cell cytosol. If *Y. pestis*-produced OMVs are capable of fusing with the host cell during infection, this raises the possibility that extracellular virulence factors of the plague bacillus may also have intracellular activities. For instance, if Pla is internalized via OMV fusion or endocytosis, the Pla protease could alter host cell function by cleaving or degrading intracellular proteins. If these targeted proteins contribute to pathogen sensing, signaling, or basic biological processes, this could explain the diverse roles of Pla during pneumonic plague beyond its effects on fibrinolysis and apoptosis.

While the release of OMVs by *Y. pestis* may be playing a natural role during host infections, it is also tempting to speculate on the use of purified OMVs as a tool to determine the specific roles of outer membrane virulence factors in the host independent of replicating, metabolically active, or secretion-competent bacteria. This could be particularly useful for the study of proteins that are otherwise intransigent to purification due to their structure or requirement for bacterial co-factors for full activity, such as Pla. Experiments using OMVs as a virulence factor “delivery system” have been performed with a variety of Gram-negative organisms including uropathogenic *E. coli* [59], *E. tarda* [63], *B. melitensis* [60], and *N. meningitidis* [71], and similar experiments with *Y. pestis* OMVs are likely to elaborate our understanding of the overall virulence strategy of this high-risk pathogen.

## Materials and Methods

### Reagents, bacterial strains and growth conditions

All reagents were obtained from Sigma-Aldrich or VWR unless otherwise indicated. All *Y. pestis* strains described in this study lack the pCD1 virulence plasmid, and bacterial strains used in this study are listed in **Table S2**. *Y. pestis* strains were routinely cultured on brain heart infusion (BHI) (Difco) agar or in liquid BHI broth at 26°C unless otherwise indicated. Media were supplemented with ampicillin (100 µg/ml) or kanamycin (50 µg/ml) as appropriate.

### Deletion of *rseA* and *lpp*

The coding sequences for *rseA* and *lpp* were deleted from *Y. pestis* by lambda red recombination following procedures previously described [25], [72]. Regions of homology upstream and downstream of the genes were amplified by PCR using the primer sets listed in **Table S3**. The kanamycin resistance cassette used for the selection of recombinants was excised using an FRT-based system as described [72].

### OMV isolation

OMVs were isolated from *Y. pestis* strains based on previously published protocols [5], [40]. Briefly, *Y. pestis* was cultured in Erlenmeyer flasks with shaking at 250 RPM for 10 h at 37°C or 26°C. For cold shock experiments, bacteria were cultured as above at 37°C and then placed in an ice slurry water bath for 1 h before proceeding with OMV isolation. Bacteria were centrifuged at 5,000 x g for 20 min and the supernatant, containing the released OMVs, was removed and filter sterilized through a 0.22 µm PES membrane (Millipore), and subsequently checked for sterility by plating on BHI agar. The sterilized supernatant was then concentrated using Centricon Plus-70 100 kDa centrifugation filters (Millipore) according to the manufacturer's recommendations. The concentrated filtrate was subjected to ultracentrifugation at 180,000 x g for 2 h at 4°C. The pelleted fraction containing OMVs was resuspended in 45% Optiprep solution, 10 mM HEPES, 0.85% NaCl, pH 7.4, and OMVs were subjected to density gradient centrifugation (40%, 35%, 30%, 25%, 20% Optiprep/Tris solutions) for 16 h at 100,000 x g at 4°C. Fractions were dialyzed against 50 mM Tris-HCl, pH 6.9 and analyzed for OMV recovery.

### OMV protein quantification

To determine the total protein abundance associated with OMVs, the Bradford Assay (Bio-Rad) was performed according to the manufacturer's commendations as described previously for quantifying OMV abundance [9], [63]. For those experiments in which OMV preparations from different conditions and/or strains were compared, OMV protein abundance was normalized to the optical density (OD_620_) of the bacterial culture at the time of harvest.

### Immunoblot analyses

The presence of Caf1, Ail, Pla, Hfq, and RpoA in OMV preparations were determined by immunoblot. Bacterial whole cell lysates were prepared by sonication as previously described [73]. OMVs or lysates (20 µg each) were mixed with reducing sample buffer (10% glycerol, 100 mM Tris-HCl, pH 6.8, 2% sodium dodecyl sulfate (SDS), 0.02% bromophenol blue, 5% β-mercaptoethanol) and separated by SDS-PAGE. Proteins were transferred to nitrocellulose membranes for immunblot analyses with antibodies against Pla [74], Ail (Eric Krukonis, University of Detroit Mercy School of Dentistry), Hfq [53], Caf1 (Abcam), and RpoA (Melanie Marketon, Indiana University).

### Electron microscopy

For scanning and transmission electron microscopy of *Y. pestis* or purified OMVs, bacteria were cultured for 6 h at 37°C or OMVs were isolated as above. For transmission electron microscopy, 10 µl of each preparation were spotted on nickel grids and incubated at room temperature for 30 min. The grids were then dried, and a solution of 2% formaldehyde/0.5% glutaraldehyde was applied for 15 min. Grids were then rinsed with PBS and negatively stained using 1% uranyl acetate for one min. For immune-gold labeling of OMVs, prior to fixation the Caf1 antibody was incubated with OMVs for 30 min and then washed 3 x for 10 min each with PBS. Secondary antibody conjugated to 6 mM gold beads (Invitrogen) was incubated with OMVs for 30 min and then washed 3 x with PBS followed by negative staining as described above. Images were obtained using the FEI Tecnai Spirit G2 microscopy. For scanning electron microscopy, samples were prepared as described, fixed with 4% paraformaldehyde/1% glutaraldehyde for 30 min followed by sequential dehydration with 20%, 40%, 60%, 80%, 95%, and 3×100% ethanol for 10 min each. Dehydrated samples were sputter-coated using the Baltec coating system and imaged on the JEOL Neo Scope Benchtop SEM.

### Plasminogen activation assay

Assessment of plg activation by *Y. pestis* bacteria, culture supernatants, or OMVs was performed as previously described [25]. Briefly, bacteria (8×10^6^ CFU, cultured in BHI at 37°C for 6 h), 0.22 µm-filtered culture supernatant, 100 kDa-filtered culture supernatant (filtrate), or increasing concentrations of OMVs were incubated with purified human glu-plg (Hematologic Technologies) (4 µg) and the chromogenic substrate D-AFK-ANSNH-iC_4_H_9_-2HBr (SN5; Haematologic) (50 µM) in a total volume of 200 µl PBS. Reaction mixtures were incubated in triplicate for 3 h at 37°C, and the absorbance at 460 nm was measured every 10–11 min in a Molecular Devices SpectraMax M5 fluorescence microplate reader.

### α2-antiplasmin degradation assay

Purified OMVs (100 µg) or *Y. pestis* bacteria (1×10^8^ CFU, cultured in BHI broth for 6 hours at 37°C) were incubated with active α2-antiplasmin (1 µg, Abcam) at 37°C. At various times, bacteria were removed by centrifugation and proteins contained within the supernatant or the OMV-containing samples were precipitated with 10% trichloroacetic acid and resuspended in an excess of sample buffer. Samples were then separated by SDS-PAGE and transferred to nitrocellulose membranes for analysis with an antibody to α2-antiplasmin (Abcam).

### ECM binding assay

To test OMV binding to various ECM components, purified BSA, Matrigel, laminin, collagen, or fibronectin (50 µg each) were added in triplicate to the wells of a 96-well plate overnight at 4°C. Unbound ECM components were removed and the wells washed 3 x with PBS. OMVs (50 µg) were then added to the wells for 18 h at 4°C. The wells were subsequently washed 3 x with PBS and then incubated with an anti-Caf1 antibody for 4 h (1∶2,000 dilution). Wells were washed 3 x with PBS, incubated for one h with a FITC-conjugated secondary antibody, washed 3 x with PBS, and then 100 µl of PBS was added. Fluorescence was measured on a Tecan Safire^2^ spectrophotometer with an excitation wavelength of 488 nm and an emission wavelength of 519 nm. Results are presented as fold change compared to the BSA wells.

### LC-MS/MS analysis

OMVs were isolated as described and proteins were denatured at 50°C with 8 M urea for 60 min. After denaturation, proteins were reduced by adding DTT to a final concentration of 1 mM and incubating at 50°C for 15 min, and subsequently alkylated by adding iodoacetamide to a final concentration of 10 mM and incubating in the dark at room temperature for 15 min. The protein sample was then diluted by the addition of ammonium bicarbonate (100 mM) to a final concentration of 1 M urea and digested with trypsin at 37°C overnight. Samples were desalted using reverse phase C18 spin columns (Thermo Fisher Scientific), and the peptides were concentrated to dryness *in vaccuo*. After drying, the peptides were suspended in 5% acetonitrile and 0.1% formic acid, loaded directly onto a 15 cm-long, 75 µM reversed-phase capillary column (ProteoPep II C18, 300 Å, 5 µm size, New Objective), and separated with a 200 min gradient from 5% acetonitrile to 100% acetonitrile on a Proxeon Easy n-LC II (Thermo Scientific). The peptides were eluted into an LTQ Orbitrap Velos mass spectrometer (Thermo Scientific) with electrospray ionization at 350 nl/minute flow rate. The mass spectrometer was operated in data-dependent mode, and for each MS1 precursor ion scan the 10 most intense ions were selected from fragmentation by collision-induced dissociation. The other parameters for mass spectrometry analysis included: resolution of MS1 set at 60,000; normalized collision energy 35%; activation time 10 ms; isolation width 1.5; and +4 and higher charge states were rejected.

The data were processed using Proteome Discoverer (version 1.4, Thermo Scientific) and searched using embedded sequest HT search engine. The data were searched against the reference proteome of *Y. pestis* downloaded from Uniprot.org. The other parameters were as follows: (i) enzyme specificity: trypsin; (ii) fixed modification: cysteine carbamidomethylation; (iv) variable modification: methionine oxidation and N-terminal acetylation; (v) precursor mass tolerance: ±10 ppm; and (vi) fragment ion mass tolerance: ±0.8 Da. All the spectra were searched against target/decoy databases and targeted false discovery rate of 1% was set to achieve high confidence assignment of peptides. Protein grouping was enabled in Proteome discoverer and proteins were grouped to satisfy the rule of parsimony. Further, in the final protein list, protein identification was considered only valid if supported by minimum of two peptides of which at least one has to be unique. The subcellular localization of identified proteins was predicted using PSORTb version 3.0 (http://www.psort.org).

### Statistical analysis

Statistical analysis were performed using GraphPad Prism 5.0. For comparison between two groups a two-tailed student's t-test was performed. For comparison of multiple groups a two-way ANOVA was performed with a Bonferroni post-test. In all cases, significance was set to a p value of <0.05.

## Supporting Information

Table S1 Proteins associated with *Yersinia pestis* OMVs identified by LC-MS/MS.
(XLSX)

Table S2 Bacterial strains used in this study.
(DOCX)

Table S3 Oligonucleotides used in this study.
(DOCX)

## References

[1] Kesty NC, Kuehn MJ (2004) Incorporation of heterologous outer membrane and periplasmic proteins into *Escherichia coli* outer membrane vesicles. J Biol Chem 279: 2069–2076. 14578354 10.1074/jbc.M307628200 PMC3525464

[2] Parker H, Keenan JI (2012) Composition and function of *Helicobacter pylori* outer membrane vesicles. Microbes Infect 14: 9–16. 21911076 10.1016/j.micinf.2011.08.007

[3] Horstman AL, Kuehn MJ (2000) Enterotoxigenic *Escherichia coli* secretes active heat-labile enterotoxin via outer membrane vesicles. J Biol Chem 275: 12489–12496. 10777535 10.1074/jbc.275.17.12489 PMC4347834

[4] Bauman SJ, Kuehn MJ (2006) Purification of outer membrane vesicles from *Pseudomonas aeruginosa* and their activation of an IL-8 response. Microbes Infect 8: 2400–2408. 16807039 10.1016/j.micinf.2006.05.001 PMC3525494

[5] Kulp A, Kuehn MJ (2010) Biological functions and biogenesis of secreted bacterial outer membrane vesicles. Annu Rev Microbiol 64: 163–184. 20825345 10.1146/annurev.micro.091208.073413 PMC3525469

[6] Schertzer JW, Whiteley M (2012) A bilayer-couple model of bacterial outer membrane vesicle biogenesis. mBio 3: e00297–00211. 22415005 10.1128/mBio.00297-11 PMC3312216

[7] Schooling SR, Beveridge TJ (2006) Membrane vesicles: an overlooked component of the matrices of biofilms. J Bacteriol 188: 5945–5957. 16885463 10.1128/JB.00257-06 PMC1540058

[8] Kuehn MJ, Kesty NC (2005) Bacterial outer membrane vesicles and the host-pathogen interaction. Genes Dev 19: 2645–2655. 16291643 10.1101/gad.1299905

[9] Wai SN, Takade A, Amako K (1995) The release of outer membrane vesicles from the strains of enterotoxigenic *Escherichia coli* . Microbiol Immunol 39: 451–456. 8569529 10.1111/j.1348-0421.1995.tb02228.x

[10] Lai CH, Listgarten MA, Hammond BF (1981) Comparative ultrastructure of leukotoxic and non-leukotoxic strains of *Actinobacillus actinomycetemcomitans* . J Periodontal Res 16: 379–389. 6459437 10.1111/j.1600-0765.1981.tb00989.x

[11] Horstman AL, Kuehn MJ (2002) Bacterial surface association of heat-labile enterotoxin through lipopolysaccharide after secretion via the general secretory pathway. J Biol Chem 277: 32538–32545. 12087095 10.1074/jbc.M203740200 PMC4391702

[12] Tan TT, Morgelin M, Forsgren A, Riesbeck K (2007) *Haemophilus influenzae* survival during complement-mediated attacks is promoted by *Moraxella catarrhalis* outer membrane vesicles. J Infect Dis 195: 1661–1670. 17471436 10.1086/517611

[13] Hellman J, Warren HS (2001) Outer membrane protein A (OmpA), peptidoglycan-associated lipoprotein (PAL), and murein lipoprotein (MLP) are released in experimental Gram-negative sepsis. J Endotoxin Res 7: 69–72. 11521086

[14] Fiocca R, Necchi V, Sommi P, Ricci V, Telford J, et al (1999) Release of *Helicobacter pylori* vacuolating cytotoxin by both a specific secretion pathway and budding of outer membrane vesicles. Uptake of released toxin and vesicles by gastric epithelium. J Pathol 188: 220–226. 10398168 10.1002/(SICI)1096-9896(199906)188:2<220::AID-PATH307>3.0.CO;2-C

[15] Gankema H, Wensink J, Guinee PA, Jansen WH, Witholt B (1980) Some characteristics of the outer membrane material released by growing enterotoxigenic *Escherichia coli* . Infect Immun 29: 704–713. 7011982 10.1128/iai.29.2.704-713.1980 PMC551183

[16] Manning AJ, Kuehn MJ (2011) Contribution of bacterial outer membrane vesicles to innate bacterial defense. BMC Microbiol 11: 258. 22133164 10.1186/1471-2180-11-258 PMC3248377

[17] Butler T (2013) Plague gives surprises in the first decade of the 21st century in the United States and worldwide. Am J Trop Med Hyg 89: 788–793. 24043686 10.4269/ajtmh.13-0191 PMC3795114

[18] Schiano CA, Lathem WW (2012) Post-transcriptional regulation of gene expression in *Yersinia* species. Front Cell Infect Microbiol 2: 129. 23162797 10.3389/fcimb.2012.00129 PMC3493969

[19] Marceau M (2005) Transcriptional regulation in *Yersinia*: an update. Curr Issues Mol Biol 7: 151–177. 16053248

[20] Hinnebusch BJ, Fischer ER, Schwan TG (1998) Evaluation of the role of the *Yersinia pestis* plasminogen activator and other plasmid-encoded factors in temperature-dependent blockage of the flea. J Infect Dis 178: 1406–1415. 9780262 10.1086/314456

[21] Sodeinde OA, Subrahmanyam YV, Stark K, Quan T, Bao Y, et al (1992) A surface protease and the invasive character of plague. Science 258: 1004–1007. 1439793 10.1126/science.1439793

[22] Burrows TW (1956) An antigen determining virulence in *Pasteurella pestis* . Nature 177: 426–427. 10.1038/177426b0 13309325

[23] Cornelis GR (2002) The *Yersinia* Ysc-Yop virulence apparatus. Int J Med Microbiol 291: 455–462. 11890544 10.1078/1438-4221-00153

[24] Cowan C, Jones HA, Kaya YH, Perry RD, Straley SC (2000) Invasion of epithelial cells by *Yersinia pestis*: evidence for a *Y. pestis*-specific invasin. Infect Immun 68: 4523–4530. 10899851 10.1128/iai.68.8.4523-4530.2000 PMC98364

[25] Lathem WW, Price PA, Miller VL, Goldman WE (2007) A plasminogen-activating protease specifically controls the development of primary pneumonic plague. Science 315: 509–513. 17255510 10.1126/science.1137195

[26] Sebbane F, Jarrett CO, Gardner D, Long D, Hinnebusch BJ (2006) Role of the *Yersinia pestis* plasminogen activator in the incidence of distinct septicemic and bubonic forms of flea-borne plague. Proc Natl Acad Sci U S A 103: 5526–5530. 16567636 10.1073/pnas.0509544103 PMC1414629

[27] Sodeinde OA, Goguen JD (1989) Nucleotide sequence of the plasminogen activator gene of *Yersinia pestis*: relationship to *ompT* of *Escherichia coli* and gene E of *Salmonella typhimurium* . Infect Immun 57: 1517–1523. 2651310 10.1128/iai.57.5.1517-1523.1989 PMC313308

[28] Kukkonen M, Lahteenmaki K, Suomalainen M, Kalkkinen N, Emody L, et al (2001) Protein regions important for plasminogen activation and inactivation of alpha2-antiplasmin in the surface protease Pla of *Yersinia pestis* . Mol Microbiol 40: 1097–1111. 11401715 10.1046/j.1365-2958.2001.02451.x

[29] Caulfield AJ, Walker ME, Gielda LM, Lathem WW (2014) The Pla protease of *Yersinia pestis* degrades Fas ligand to manipulate host cell death and inflammation. Cell Host Microbe 15: 424–434. 24721571 10.1016/j.chom.2014.03.005 PMC4020149

[30] Caulfield AJ, Lathem WW (2012) Substrates of the plasminogen activator protease of *Yersinia pestis* . Adv Exp Med Biol 954: 253–260. 22782771 10.1007/978-1-4614-3561-7_32 PMC3513919

[31] Beesley ED, Brubaker RR, Janssen WA, Surgalla MJ (1967) Pesticins. 3. Expression of coagulase and mechanism of fibrinolysis. J Bacteriol 94: 19–26. 6027989 10.1128/jb.94.1.19-26.1967 PMC251865

[32] Lahteenmaki K, Virkola R, Saren A, Emody L, Korhonen TK (1998) Expression of plasminogen activator Pla of *Yersinia pestis* enhances bacterial attachment to the mammalian extracellular matrix. Infect Immun 66: 5755–5762. 9826351 10.1128/iai.66.12.5755-5762.1998 PMC108727

[33] Kienle Z, Emody L, Svanborg C, O′Toole PW (1992) Adhesive properties conferred by the plasminogen activator of *Yersinia pestis* . J Gen Microbiol 138 Pt 8: 1679–1687. 10.1099/00221287-138-8-1679 1527508

[34] Eren E, van den Berg B (2012) Structural basis for activation of an integral membrane protease by lipopolysaccharide. J Biol Chem 287: 23971–23976. 22645135 10.1074/jbc.M112.376418 PMC3390672

[35] Eren E, Murphy M, Goguen J, van den Berg B (2010) An active site water network in the plasminogen activator pla from *Yersinia pestis* . Structure 18: 809–818. 20637417 10.1016/j.str.2010.03.013

[36] Pouillot F, Derbise A, Kukkonen M, Foulon J, Korhonen TK, et al (2005) Evaluation of O-antigen inactivation on Pla activity and virulence of *Yersinia pseudotuberculosis* harbouring the pPla plasmid. Microbiology 151: 3759–3768. 16272397 10.1099/mic.0.28274-0

[37] Sodeinde OA, Sample AK, Brubaker RR, Goguen JD (1988) Plasminogen activator/coagulase gene of *Yersinia pestis* is responsible for degradation of plasmid-encoded outer membrane proteins. Infect Immun 56: 2749–2752. 2843471 10.1128/iai.56.10.2749-2752.1988 PMC259639

[38] Kolodziejek AM, Caplan AB, Bohach GA, Paszczynski AJ, Minnich SA, et al (2013) Physiological levels of glucose induce membrane vesicle secretion and affect the lipid and protein composition of *Yersinia pestis* cell surfaces. Appl Environ Microbiol 79: 4509–4514. 23686263 10.1128/AEM.00675-13 PMC3697494

[39] Ellis TN, Leiman SA, Kuehn MJ (2010) Naturally produced outer membrane vesicles from *Pseudomonas aeruginosa* elicit a potent innate immune response via combined sensing of both lipopolysaccharide and protein components. Infect Immun 78: 3822–3831. 20605984 10.1128/IAI.00433-10 PMC2937433

[40] McBroom AJ, Kuehn MJ (2007) Release of outer membrane vesicles by Gram-negative bacteria is a novel envelope stress response. Mol Microbiol 63: 545–558. 17163978 10.1111/j.1365-2958.2006.05522.x PMC1868505

[41] Chutkan H, Macdonald I, Manning A, Kuehn MJ (2013) Quantitative and qualitative preparations of bacterial outer membrane vesicles. Methods Mol Biol 966: 259–272. 23299740 10.1007/978-1-62703-245-2_16 PMC4317262

[42] Pieper R, Huang ST, Robinson JM, Clark DJ, Alami H, et al (2009) Temperature and growth phase influence the outer-membrane proteome and the expression of a type VI secretion system in *Yersinia pestis* . Microbiology 155: 498–512. 19202098 10.1099/mic.0.022160-0

[43] Lane MC, Lenz JD, Miller VL (2013) Proteolytic processing of the *Yersinia pestis* YapG autotransporter by the omptin protease Pla and the contribution of YapG to murine plague pathogenesis. J Med Microbiol 62: 1124–1134. 23657527 10.1099/jmm.0.056275-0 PMC3749520

[44] Yonezawa H, Osaki T, Woo T, Kurata S, Zaman C, et al (2011) Analysis of outer membrane vesicle protein involved in biofilm formation of *Helicobacter pylori* . Anaerobe 17: 388–390. 21515394 10.1016/j.anaerobe.2011.03.020

[45] Schwechheimer C, Sullivan CJ, Kuehn MJ (2013) Envelope control of outer membrane vesicle production in Gram-negative bacteria. Biochemistry 52: 3031–3040. 23521754 10.1021/bi400164t PMC3731998

[46] Schwechheimer C, Kuehn MJ (2013) Synthetic effect between envelope stress and lack of outer membrane vesicle production in *Escherichia coli* . J Bacteriol 195: 4161–4173. 23852867 10.1128/JB.02192-12 PMC3754735

[47] Macdonald IA, Kuehn MJ (2013) Stress-induced outer membrane vesicle production by *Pseudomonas aeruginosa* . J Bacteriol 195: 2971–2981. 23625841 10.1128/JB.02267-12 PMC3697536

[48] Galindo CL, Sha J, Moen ST, Agar SL, Kirtley ML, et al. (2010) Comparative global gene expression profiles of wild-type *Yersinia pestis* CO92 and its braun lipoprotein mutant at flea and human body temperatures. Comp Funct Genomics: 342168. 10.1155/2010/342168 PMC2873655 20508723

[49] Phadtare S (2004) Recent developments in bacterial cold-shock response. Curr Issues Mol Biol 6: 125–136. 15119823

[50] Rowley G, Spector M, Kormanec J, Roberts M (2006) Pushing the envelope: extracytoplasmic stress responses in bacterial pathogens. Nat Rev Microbiol 4: 383–394. 16715050 10.1038/nrmicro1394

[51] Missiakas D, Mayer MP, Lemaire M, Georgopoulos C, Raina S (1997) Modulation of the *Escherichia coli* sigmaE (RpoE) heat-shock transcription-factor activity by the RseA, RseB and RseC proteins. Mol Microbiol 24: 355–371. 9159522 10.1046/j.1365-2958.1997.3601713.x

[52] Alba BM, Gross CA (2004) Regulation of the *Escherichia coli* sigma-dependent envelope stress response. Mol Microbiol 52: 613–619. 15101969 10.1111/j.1365-2958.2003.03982.x

[53] Schiano CA, Bellows LE, Lathem WW (2010) The small RNA chaperone Hfq is required for the virulence of *Yersinia pseudotuberculosis* . Infect Immun 78: 2034–2044. 20231416 10.1128/IAI.01046-09 PMC2863511

[54] Lathem WW, Schroeder JA, Bellows LE, Ritzert JT, Koo JT, et al (2014) Posttranscriptional regulation of the *Yersinia pestis* cyclic AMP receptor protein Crp and impact on virulence. mBio 5: e01038–01013. 24520064 10.1128/mBio.01038-13 PMC3950509

[55] Geng J, Song Y, Yang L, Feng Y, Qiu Y, et al (2009) Involvement of the post-transcriptional regulator Hfq in *Yersinia pestis* virulence. PLoS One 4: e6213. 19593436 10.1371/journal.pone.0006213 PMC2704395

[56] Nikaido H, Bavoil P, Hirota Y (1977) Outer membranes of gram-negative bacteria. XV. Transmembrane diffusion rates in lipoprotein-deficient mutants of *Escherichia coli* . J Bacteriol 132: 1045–1047. 200601 10.1128/jb.132.3.1045-1047.1977 PMC235612

[57] Cascales E, Bernadac A, Gavioli M, Lazzaroni JC, Lloubes R (2002) Pal lipoprotein of *Escherichia coli* plays a major role in outer membrane integrity. J Bacteriol 184: 754–759. 11790745 10.1128/JB.184.3.754-759.2002 PMC139529

[58] Kesty NC, Mason KM, Reedy M, Miller SE, Kuehn MJ (2004) Enterotoxigenic *Escherichia coli* vesicles target toxin delivery into mammalian cells. EMBO J 23: 4538–4549. 15549136 10.1038/sj.emboj.7600471 PMC533055

[59] Davis JM, Carvalho HM, Rasmussen SB, O′Brien AD (2006) Cytotoxic necrotizing factor type 1 delivered by outer membrane vesicles of uropathogenic *Escherichia coli* attenuates polymorphonuclear leukocyte antimicrobial activity and chemotaxis. Infect Immun 74: 4401–4408. 16861625 10.1128/IAI.00637-06 PMC1539604

[60] Avila-Calderon ED, Lopez-Merino A, Jain N, Peralta H, Lopez-Villegas EO, et al (2012) Characterization of outer membrane vesicles from *Brucella melitensis* and protection induced in mice. Clin Dev Immunol 2012: 352493. 22242036 10.1155/2012/352493 PMC3254011

[61] Tsang TM, Felek S, Krukonis ES (2010) Ail binding to fibronectin facilitates *Yersinia pestis* binding to host cells and Yop delivery. Infect Immun 78: 3358–3368. 20498264 10.1128/IAI.00238-10 PMC2916272

[62] Ismail S, Hampton MB, Keenan JI (2003) *Helicobacter pylori* outer membrane vesicles modulate proliferation and interleukin-8 production by gastric epithelial cells. Infect Immun 71: 5670–5675. 14500487 10.1128/IAI.71.10.5670-5675.2003 PMC201067

[63] Park SB, Jang HB, Nho SW, Cha IS, Hikima J, et al (2011) Outer membrane vesicles as a candidate vaccine against edwardsiellosis. PLoS One 6: e17629. 21408115 10.1371/journal.pone.0017629 PMC3050902

[64] Lee EY, Choi DS, Kim KP, Gho YS (2008) Proteomics in gram-negative bacterial outer membrane vesicles. Mass Spectrom Rev 27: 535–555. 18421767 10.1002/mas.20175

[65] Lee EY, Bang JY, Park GW, Choi DS, Kang JS, et al (2007) Global proteomic profiling of native outer membrane vesicles derived from *Escherichia coli* . Proteomics 7: 3143–3153. 17787032 10.1002/pmic.200700196

[66] Pieper R, Huang ST, Clark DJ, Robinson JM, Alami H, et al (2009) Integral and peripheral association of proteins and protein complexes with *Yersinia pestis* inner and outer membranes. Proteome Sci 7: 5. 19228400 10.1186/1477-5956-7-5 PMC2663777

[67] Post DM, Zhang D, Eastvold JS, Teghanemt A, Gibson BW, et al (2005) Biochemical and functional characterization of membrane blebs purified from *Neisseria meningitidis* serogroup B. J Biol Chem 280: 38383–38394. 16103114 10.1074/jbc.M508063200

[68] Shah S, Miller A, Mastellone A, Kim K, Colaninno P, et al (1998) Rapid diagnosis of tuberculosis in various biopsy and body fluid specimens by the AMPLICOR *Mycobacterium tuberculosis* polymerase chain reaction test. Chest 113: 1190–1194. 9596293 10.1378/chest.113.5.1190

[69] Dorward DW, Schwan TG, Garon CF (1991) Immune capture and detection of *Borrelia burgdorferi* antigens in urine, blood, or tissues from infected ticks, mice, dogs, and humans. J Clin Microbiol 29: 1162–1170. 1864935 10.1128/jcm.29.6.1162-1170.1991 PMC269963

[70] Bjerre A, Brusletto B, Rosenqvist E, Namork E, Kierulf P, et al (2000) Cellular activating properties and morphology of membrane-bound and purified meningococcal lipopolysaccharide. J Endotoxin Res 6: 437–445. 11521068

[71] Saunders NB, Brandt BL, Warren RL, Hansen BD, Zollinger WD (1998) Immunological and molecular characterization of three variant subtype P1.14 strains of *Neisseria meningitidis* . Infect Immun 66: 3218–3222. 9632588 10.1128/iai.66.7.3218-3222.1998 PMC108335

[72] Koo JT, Alleyne TM, Schiano CA, Jafari N, Lathem WW (2011) Global discovery of small RNAs in *Yersinia pseudotuberculosis* identifies *Yersinia*-specific small, noncoding RNAs required for virulence. Proc Natl Acad Sci U S A 108: E709–717. 21876162 10.1073/pnas.1101655108 PMC3174644

[73] Bellows LE, Koestler BJ, Karaba SM, Waters CM, Lathem WW (2012) Hfq-dependent, co-ordinate control of cyclic diguanylate synthesis and catabolism in the plague pathogen *Yersinia pestis* . Mol Microbiol 86: 661–674. 22924957 10.1111/mmi.12011 PMC3480973

[74] Houppert AS, Bohman L, Merritt PM, Cole CB, Caulfield AJ, et al (2013) RfaL is required for *Yersinia pestis* type III secretion and virulence. Infect Immun 81: 1186–1197. 23357388 10.1128/IAI.01417-12 PMC3639585

