# Supplementary material for: Production of Outer Membrane Vesicles by the Plague Pathogen Yersinia pestis
Source: PLoS One. 2014 Sep 8;9(9):e107002. doi: 10.1371/journal.pone.0107002 (PMC4157834; doi:10.1371/journal.pone.0107002)
Supplement: Table S2 — Bacterial strains used in this study. (DOCX) [file pone.0107002.s002.docx]

**Table S2.** Bacterial strains used in this study.

| ***Y. pestis* strain** | **Strain designation** | **Genotype and/or characteristics** | **Source or reference** |
| --- | --- | --- | --- |
| CO92 LCR- | PAN259 | pCD1- pPCP1+ pMT1+ pgm+ | Lab stock |
| CO92 LCR- ∆*pla* | PAN314 | ∆*pla* | Lab stock |
| CO92 LCR- ∆*lpp* | PAN332 | ∆*lpp* | This study |
| CO92 LCR- ∆*hfq* | PAN300 | ∆*hfq* | Schiano et al. 2010 |
| CO92 LCR- ∆*rseA* | PAN28 | ∆*rseA* | This study |
