# Supplementary material for: Production of Outer Membrane Vesicles by the Plague Pathogen Yersinia pestis
Source: PLoS One. 2014 Sep 8;9(9):e107002. doi: 10.1371/journal.pone.0107002 (PMC4157834; doi:10.1371/journal.pone.0107002)
Supplement: Table S3 — Oligonucleotides used in this study. (DOCX) [file pone.0107002.s003.docx]

**Table S3.** Oligonucleotides used in this study.

| **Oligo name** | **Sequence (5’ 🡪 3’)** |
| --- | --- |
| rseA 5' -500 | GAGCCTCGTATCCCGGTATGTG |
| rseA 3' +500 | TGGCAGTTTCGTTGCCTCATCCAG |
| P1 rseA 3'3 | GAAGCAGCTCCAGCCTACACCATGCCTAAATACCCTTTGTGTGTCCG |
| P4 rseA 5'656 | GGTCGACGGATCCCCGGAATAATGAAGCAACTTTGGTTTTCCGTCTG |
| lpp 3' +415 | CGAGACACTCATCTTGGGCGG |
| lpp 5' -498 | TAAAGGCGTAATCACTCAGTTGGT |
| lpp 3' SOE +25 | GGTCGACGGATCCCCGGAATTTTACTTCCTGAATGAAAAATGGCGC |
| lpp 5' SOE -27 | GAAGCAGCTCCAGCCTACACCATTATTAACACCGTGTAGATTAAGTT |
